# Microvascular endothelial function following cessation of long‐term oral contraceptive pill use: A case report

**DOI:** 10.1113/EP090861

**Published:** 2022-11-30

**Authors:** Casey G. Turner, Anna E. Stanhewicz, Karen E. Nielsen, Brett J. Wong

**Affiliations:** ^1^ Department of Kinesiology and Health Georgia State University Atlanta Georgia USA; ^2^ Department of Health and Human Physiology University of Iowa Iowa City Iowa USA; ^3^ Department of Population Health Sciences School of Public Health Georgia State University Atlanta Georgia USA

**Keywords:** case report, endothelium, nitric oxide, oral contraceptives, women

## Abstract

The purpose of this case report was to evaluate in vivo endothelial function and nitric oxide (NO)‐dependent vasodilatation before and after the cessation of long‐term (11–12 years) fourth‐generation oral contraceptive pill (OCP) use in one young, healthy and premenopausal woman. This retrospective analysis includes data from six experimental visits: three visits during months 133–144 of fourth‐generation OCP use and three visits 19–22 months after OCP cessation. Endothelium‐dependent and NO‐dependent vasodilatation were assessed in the cutaneous microvasculature using laser‐Doppler flowmetry, a rapid local heating protocol (39°C, 0.1°C/s) and pharmacological perfusion through intradermal microdialysis fibres. The participant had consistent medical history and lifestyle behaviours throughout both hormonal exposures. Data are presented as the mean (SD). Endothelium‐dependent vasodilatation was 42 (10)% of site‐specific maximal cutaneous vascular conductance (CVC_max_) during OCP use and 63 (10)%CVC_max_ after OCP cessation (49% increase). Nitric oxide‐dependent vasodilatation was 70 (5)% contribution of NO during OCP use and 60 (15)%NO after OCP cessation (15% reduction). Baseline blood flow was greater after OCP cessation, but maximal blood flow was reduced. Data from this case report support a substantial increase in cutaneous microvascular endothelial function assessed via local heating after cessation of long‐term use of a fourth‐generation OCP, which does not appear to be attributable to increased NO bioavailability. Overall, these data suggest an improvement in endothelial and microvascular function after the cessation of long‐term use of a fourth‐generation OCP.

## Abstract (highlights)

- **What is the main observation in this case?**
  The main observation of this case report is substantial improvement in cutaneous microvascular endothelial function after cessation of long‐term use of a fourth‐generation oral contraceptive pill. This improvement appears independent of relative changes in the contribution of nitric oxide.
- **What insights does it reveal?**
  Our findings suggest that cessation of long‐term, fourth‐generation oral contraceptive pill use improves endothelial function within 20 months of cessation.

## 1 INTRODUCTION

Oral contraceptive pill (OCP) use can be a unique risk factor for cardiovascular complications in women (Dragoman et al., 2018; Kaminski et al., 2013; Roach et al., 2015; Vinogradova et al., 2015). Women using the OCP are at increased risk for venous and arterial thromboembolism compared with naturally cycling (NC) women, but the relative risk of complications appears to vary with different OCP formulations (Dragoman et al., 2018; Roach et al., 2015; Vinogradova et al., 2015). For example, preparations including drospirenone (a fourth‐generation progestin) are associated with approximately twofold greater thromboembolic risk compared with preparations including levonorgestrel (a second‐generation progestin) (Dragoman et al., 2018; Vinogradova et al., 2015). However, previous data suggest that the risk of myocardial infarction and stroke (corresponding to arterial thromboembolism) resolves after OCP cessation (Baillargeon et al., 2005). The endothelium regulates thrombogenesis, in part through synthesis of the antithrombotic molecule nitric oxide (NO) (Daiber et al., 2017). This suggests an association between current OCP use and endothelial function.

Approximately 75–85% of women globally use OCP during their premenopausal years (Daniels et al., 2013; Margolis et al., 2007; Yusuf & Siedlecky, 2007), but current usage is estimated at 14–16% of premenopausal women (Daniels & Abma, 2020; Rotermann et al., 2015). This suggests that a large proportion of women have been exposed to post‐OCP conditions. To date, there is a lack of research assessing microvascular or endothelial function after long‐term OCP use, and there has been no direct assessment of microvascular or endothelial function after the cessation of OCP use, as highlighted recently in a review by Williams and MacDonald (2021). This might be attributable, in part, to the difficult nature of designing a prospective experiment aimed to assess OCP cessation. Therefore, this case report highlights a unique account of volitional OCP cessation after long‐term use that is both relevant and practical. The purpose of this case report was to assess the impact of OCP cessation on in vivo endothelial function and NO bioavailability. For this case report, endothelial function was assessed in the cutaneous microvasculature, which is a surrogate of systemic microvascular function (Holowatz et al., 2008).

## 2 METHODS

### 2.1 Ethical approval

This is a retrospective analysis of data from studies approved by Advarra Institutional Review Board (Columbia, MD, USA; numbers Pro00024265 and Pro00056105) and the US Food and Drug Administration (numbers IND 138231 and IND 157532). All experimental procedures conformed to the *Declaration of Helsinki*. The participant provided written and verbal consent before participating in any experimental procedures.

### 2.2 Participant information

Data were recorded from one premenopausal woman between July 2018 and September 2021, when she was 26–29 years old. She had a 12‐year history of OCP use (fourth‐generation, monophasic, 0.02 mg ethinyl estradiol and 3.0 mg drospirenone), which was discontinued volitionally in November 2019. The participant self‐identified the racial/ethnic background of herself and both biological parents as non‐Hispanic White. The participant reported health history and lifestyle behaviour information at each visit via questionnaire. She had no reported diagnosis of cardiovascular, pulmonary or metabolic disease and no reported diagnosis of any dermatological conditions. Furthermore, she was not taking any prescription medications apart from the specified OCP, indulged in regular physical activity, maintained consistent sleep and dietary behaviours and was nulliparous throughout this entire time span.

### 2.3 Experimental visits

This data set includes measurements from six experimental visits. Three experimental visits were completed during months 133–144 of OCP use. The first, second and third time points correspond, respectively, to a visit during active pills at month 133, a visit during active pills at month 134, and a visit during placebo pills at month 144 of OCP use. Three experimental visits were also completed 19–22 months after cessation of OCP use. The fourth, fifth and sixth time points refer, respectively, to NC assessments on day 4 (during the menstrual phase) of the menstrual cycle at month 19, day 16 of the menstrual cycle at month 20, and day 10 of the menstrual cycle at month 22 post‐cessation. Over the NC testing time frame, the average cycle length was 32 (1, SD) days, without a cycle lasting >40 days within the last 10 months. Endogenous sex hormone concentrations were not verified for NC visits in this retrospective analysis. Of the six experimental visits, five took place within an hour of each other (between 12.30 and 13.30 h). The remaining visit (visit 3 in the data) took place at 09.00 h.

### 2.4 Instrumentation

The participant was asked to refrain from alcohol, vigorous exercise, caffeine, and meals high in fat or sugar for ≥8 h before the experimental protocol. The participant was seated in the semi‐recumbent position, and the experimental arm (left) was positioned and secured at heart level to minimize the effect of hydrostatic pressure on blood flow. Data for this retrospective analysis were obtained from a single intradermal microdialysis fibre (CMA 31; Harvard Apparatus, Hollister, MA, USA) site on the dorsal forearm. Microdialysis fibre placement occurred as described previously (Miller et al., 2021; Turner et al., 2020; Wong et al., 2020). To control local skin temperature, a local heater unit (VHP1 heater units and VMS‐HEAT controller; Moor Instruments, Axminster, UK) was placed directly over the microdialysis membrane. An integrated laser‐Doppler probe (VP7b probes and VMS‐LDF2 monitor; Moor Instruments) was placed in the centre of the local heating unit to obtain red blood cell (RBC) flux, an index of skin blood flow, at the microdialysis site. Blood pressure was measured every 10 min from the contralateral arm using an automated brachial oscillometric device, and heart rate was derived from pulse detection (Welch Allyn Vital Signs Series 6000; Skaneatelles Falls, NY, USA). Mean arterial pressure (MAP) was calculated as one‐third pulse pressure plus diastolic pressure.

### 2.5 Experimental protocol

The microdialysis fibre was perfused with lactated Ringer solution (Baxter Healthcare, Deerfield, IL, USA) during the trauma resolution period (∼60 min), baseline and rapid local heating (described below). The local heater unit was set initially to thermoneutral (33°C), and baseline skin blood flow was measured for ∼15 min. A rapid local heating protocol was then conducted to elicit endothelium‐dependent vasodilatation, whereby local heater temperature was increased from 33 to 39°C at a rate of 0.1°C/s (Choi et al., 2014). Once a plateau in skin blood flow was achieved, 20 mM *N* ^ω^‐nitro‐l‐arginine methyl ester (l‐NAME, an NO synthase inhibitor) was perfused through the microdialysis fibre to calculate the contribution of NO to vasodilatation (Miller et al., 2021; Wong et al., 2020). Once a new plateau following l‐NAME perfusion (i.e., post‐l‐NAME plateau) was achieved, maximal vasodilatation was induced by heating the skin from 39 to 43°C and infusing 28 mM sodium nitroprusside (exogenous NO donor) (Stanhewicz et al., 2017). All solutions were perfused at a rate of 2 μl/min (Beehive Controller and Baby Bee syringe pumps; Bioanalytical Systems, West Lafayette, IN, USA). Pharmacological agents were diluted with sterile lactated Ringer solution (Smith et al., 2017) and drawn through filter needles (BD Filter Needle; Becton Dickinson, Franklin Lakes, NJ, USA).

### 2.6 Data analysis

Skin blood flow data were recorded continuously at 40 Hz using commercially available hardware and software (PowerLab 16/35 data acquisition and Lab Chart 8 software; ADInstruments, Colorado Springs, CO, USA). Cutaneous vascular conductance (CVC) was calculated as RBC flux divided by MAP and standardized to maximal vasodilatation (%CVC_max_) (Braverman et al., 1990; Cracowski et al., 2006). The following four main periods of skin blood flow data were analysed: (1) baseline; (2) plateau; (3) post‐l‐NAME plateau; and (4) maximal vasodilatation. Approximate 3 min windows of data were analysed during the experimental protocol, corresponding to each of the four phases, respectively: (1) immediately preceding the onset of the local heating protocol; (2) immediately preceding the infusion of l‐NAME; (3) immediately preceding initiation of maximal vasodilatation; and (4) before cessation of the experimental protocol. Within‐day variability of the data was similar across hormonal exposure. The percentage contribution of NO (%NO) to the plateau was calculated using plateau and post‐l‐NAME values (Wong & Fieger, 2010). All data were plotted graphically using commercially available software (GraphPad Prism v.8; GraphPad, San Diego, CA, USA). All data are presented as the mean (SD), and the percentage change was calculated.

## 3 RESULTS

**FIGURE 1:**
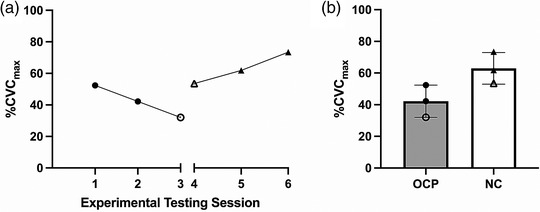
Endothelium‐dependent vasodilatation (i.e., plateau). (a) Responses at individual visits over the transition of fourth‐generation OCP cessation. (b) Mean values during OCP use and after cessation. Circles indicate assessments during months 133–144 of fourth‐generation OCP use (*n* = 3 experimental visits). Triangles indicate assessments during months 19–22 after cessation of OCP use (*n* = 3 experimental visits). Open symbols indicate visits during the placebo pill OCP phase or during the menstrual phase of the natural menstrual cycle. Filled symbols indicate visits during the active pill OCP phase or outside of the menstrual phase of the natural menstrual cycle. Abbreviations: %CVC_max_, percentage of site‐specific maximal cutaneous vascular conductance; NC, naturally cycling; OCP, oral contraceptive pill

**FIGURE 2:**
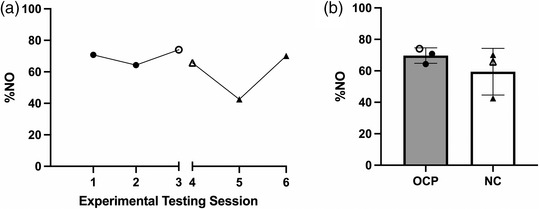
Nitric oxide‐dependent vasodilatation. (a) Responses at individual visits over the transition of fourth‐generation OCP cessation. (b) Mean values during OCP use and after cessation. Circles indicate assessments during months 133–144 of fourth‐generation OCP use (*n* = 3 experimental visits). Triangles indicate assessments during months 19–22 following cessation of OCP use (*n* = 3 experimental visits). Open symbols indicate visits during the placebo pill OCP phase or during the menstrual phase of the natural menstrual cycle. Filled symbols indicate visits during the active pill OCP phase or outside of the menstrual phase of the natural menstrual cycle. Abbreviations: %NO, percentage of endothelium‐dependent vasodilatation attributable to nitric oxide; NC, naturally cycling; OCP, oral contraceptive pill

**FIGURE 3:**
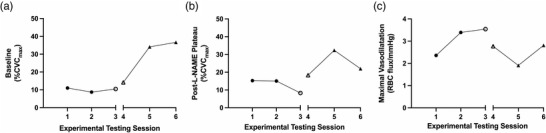
Responses at individual visits for additional phases of the local heating response: (a) baseline; (b) post‐l‐NAME plateau; and (c) maximal vasodilatation. Circles indicate assessments during months 133–144 of fourth‐generation OCP use (*n* = 3 experimental visits). Triangles indicate assessments during months 19–22 after cessation of OCP use (*n* = 3 experimental visits). Open symbols indicate visits during the placebo pill OCP phase or during the menstrual phase of the natural menstrual cycle. Filled symbols indicate visits during the active pill OCP phase or outside of the menstrual phase of the natural menstrual cycle. Abbreviations: %CVC_max_, percentage of site‐specific maximal cutaneous vascular conductance; l‐NAME, *N* ^ω^‐nitro‐l‐arginine methyl ester; NC, naturally cycling; OCP, oral contraceptive pill; RBC, red blood cell

Throughout the study period, the participant reported consistent health history information and indices of lifestyle behaviours, including self‐reported quantity and intensity of physical activity, quantity and quality of sleep, and basic dietary habits (intake of salt, fat, caffeine, alcohol, etc.). Information on participant characteristics across the OCP and NC exposures is displayed in Table 1. Plateau blood flow (i.e., endothelium‐dependent vasodilatation) averaged 42 (10)%CVC_max_ during OCP use and 63 (10)%CVC_max_ after OCP cessation (49% increase; Figure 1). Nitric oxide‐dependent vasodilatation was 70 (5)%NO during OCP use and 60 (15)%NO after OCP cessation (15% reduction; Figure 2). During OCP use, baseline blood flow averaged 10 (1)%CVC_max_, and after cessation of OCP it averaged 28 (12)%CVC_max_ (181% increase; Figure 3a). The post‐l‐NAME plateau was 13 (4)%CVC_max_ during OCP use and 24(7)%CVC_max_ after OCP cessation (88% increase; Figure 3b). Maximal vasodilatation averaged 3.10 (0.64) RBC flux/mmHg during OCP use and 2.50 (0.51) RBC flux/mmHg after OCP cessation (19% reduction; Figure 3c).

**TABLE 1:** Participant characteristics

**Characteristic**	**OCP** **(*n* = 3 visits)**	**NC** **(*n* = 3 visits)**
Age (years)	26–27	29
Height (m)	1.57	1.57
Weight (kg)	55.5 (1.3)	56.1 (0.8)
Body mass index (kg/m^2^)	22.4 (0.5)	22.6 (0.3)
Resting heart rate (beats/min)	68 (3)	68 (4)
Systolic blood pressure (mmHg)	108 (4)	107 (6)
Diastolic blood pressure (mmHg)	67 (3)	68 (5)
Mean arterial pressure (mmHg)	81 (4)	81 (5)

*Note*: The OCP used by this individual was a monophasic, fourth‐generation OCP containing 0.02 mg of ethinyl estradiol and 3.0 mg of drospirenone. Data are presented as the mean (SD).

Abbreviations: NC, naturally cycling; OCP, oral contraceptive pills.

## 4 DISCUSSION

The key findings from this case report are as follows: (1) there was a substantial increase in endothelium‐dependent vasodilatation (Figure 1) after cessation of long‐term, fourth‐generation OCP use; and (2) NO‐dependent vasodilatation decreased after OCP cessation, but this was driven largely by the response at visit 5 (Figure 2). Overall, NO‐dependent vasodilatation was generally consistent before and after cessation of fourth‐generation OCP use, suggesting that other mechanisms might be involved.

### 4.1 Endothelium‐dependent vasodilatation

There was a consistent increase in endothelium‐dependent vasodilatation after the cessation of long‐term use of a fourth‐generation OCP (49% observed increase). This finding was in the absence of any evident changes in physical health (i.e., no changes in body mass index, haemodynamics, medical history, lifestyle habits, etc.) between the two hormonal exposures. Notably, the measured endothelium‐dependent vasodilatation of 42%CVC_max_, such as during fourth‐generation OCP use in this case report, is similar to that seen in populations known to be at increased risk of cardiovascular disease, such as non‐Hispanic Black or African American adults (Hurr et al., 2018; Miller et al., 2021; Patik et al., 2018; Wong et al., 2020). This might indicate endothelial dysfunction and acutely increased cardiovascular risk in women currently using OCP. However, it is unclear from this case report whether the observed increase in endothelial function after OCP cessation is reflective of effects of long‐term OCP use in general or whether there are specific implications for fourth‐generation OCPs or drospirenone.

It is currently unclear which signalling pathways might mediate this difference. Data from this case report suggest that differences in endothelium‐dependent vasodilatation are not explained fully by changes in NO. Oral contraceptive pill use has been associated with increases in oxidative stress and inflammation (Cauci et al., 2016, 2017, 2021), which might activate pathways detrimental to endothelial function. Further assessment of mechanistic changes is warranted, because some findings suggest that OCP use at any time can impact future cardiovascular health (Lee et al., 2022).

### 4.2 Post‐l‐NAME plateau and NO‐dependent vasodilatation

The contribution of NO was consistent through visits during OCP use, including either placebo or active pill days. It is unclear whether this is a feature of fourth‐generation progestins, drospirenone itself, or universal across combination OCPs. Absolute sensitivity to inhibition of NO synthase was greater during OCP use (i.e., lower post‐l‐NAME plateau; Figure 3b), and relative NO‐dependent vasodilatation was greater (Figure 2) during OCP use. Nitric oxide‐dependent vasodilatation at two of the three NC data points (visits 4 and 6, average 68%NO) was similar to NO‐dependent vasodilatation during the three visits during OCP use (average 70%NO). However, in this data set, the measurement from NC visit 5 (day 16 of month 20 post‐cessation) was markedly lower than the other two NC measurements (43%NO). Therefore, it is unclear whether the finding of a 15% reduction in NO‐dependent vasodilatation has relevance within this case report or whether it would be in a larger sample size of a similar cohort. Furthermore, it is not clear why NO‐dependent vasodilatation differed so greatly at this time point compared with others. Reduced NO‐dependent vasodilatation is unlikely to be explained by circadian variations, because the experimental visit with the lowest %NO took place at roughly the same time of day as four of the other experimental visits.

### 4.3 Baseline and maximal blood flow

There was an observed 181% increase in baseline blood flow after OCP cessation (Figure 3a), more strongly exhibited during the NC visits not during the menstrual phase. Previous data suggest an increase in basal NO release with the administration of ethinyl estradiol (Arnal et al., 1996). Taken together with our data, this suggests that other facets of combination OCP might compromise basal vascular tone, either functionally or structurally. There was also a 19% reduction in average maximal blood flow after OCP cessation (Figure 3c). In accordance with assumptions in the field, this might be related to structural changes in the microvasculature (Holowatz & Kenney, 2007a, 2007b). This case report reveals no further context for the reason for increased baseline blood flow or decreased maximal blood flow after fourth‐generation OCP cessation, but these findings suggest that further investigation is warranted.

### 4.4 Strengths and limitations

The strengths of this case report include the following. First, importantly, it would be difficult logistically to complete an adequately powered, prospective study aimed at investigating outcomes after OCP cessation after long‐term use, especially to the extent demonstrated in this case report (11–12 years of OCP use). This highlights the strength of adopting a case report approach to yield insights about multiple gaps within the literature (Williams & MacDonald, 2021). Second, this is the first assessment of endothelial function over the transition from long‐term OCP use to NC. Third, this case report includes assessments at multiple time points before and after OCP cessation. Fourth, the health status of the participant and the type of OCP used were consistent over the entire span of study.

Limitations of this case report are as follows. First, there is low external validity, given that this is a singular account. Second, there is a lack of assessment closer to OCP cessation. Third, hormonal phase was not controlled across visits. During each hormonal exposure, there were visits during estimated low‐hormone phases (i.e., OCP placebo pills and NC menstrual phase) and during phases estimated to have higher hormone levels (i.e., OCP active pills and NC non‐menstrual phase); therefore, the mean ± SD data might not accurately represent function specific to certain hormone phases during either exposure. Fourth, given that this was a retrospective analysis from previous studies, we did not have blood samples available to analyse for blood lipids, glucose or sex hormones. Likewise, we did not use ovulation test kits to define the NC phase objectively. Regardless of the noted limitations, this case report highlights a distinct gap in the current literature regarding vascular effects over the transition of OCP cessation that is relevant to a large proportion of the population.

## 5 CONCLUSION

In this case report, we provide evidence of substantially increased endothelial function after the cessation of long‐term use of a fourth‐generation OCP. We also provide evidence suggesting that this is not entirely attributable to changes in activity of the NO pathway; therefore, further evaluation is warranted to determine the mechanisms underlying endothelial function during OCP use. Additionally, long‐term, fourth‐generation OCP use appears to alter maximal and basal blood flow compared with the natural menstrual cycle. Data from this case report also indicate that repeated‐measures designs might be most effective to evaluate the effect of OCP on endothelial function, because the context might be lost when completing cross‐sectional analysis of independent groups. Overall, these data indicate alterations in endothelial and microvascular function and, possibly, structure after cessation of long‐term, fourth‐generation OCP use.

## AUTHOR CONTRIBUTIONS

All data were collected in the Department of Kinesiology & Health at Georgia State University. Casey G. Turner was responsible for conception of the work, acquistion, analysis and interpretation of the work, and drafting of the work. Anna E. Stanhewicz was responsible for analysis and interpretation of the work and revising the work for important intellecutal content. Karen E. Nielsen was responsible for analysis of the work and revising the work for important intellectual content. Brett J. Wong was responsible for conception and design of the work, interpretation of the work, and revising the work for important intellectual content. All authors approved the final version of the manuscript. All authors agree to be accountable for all aspects of the work. All persons designated as authors qualify for authorship, and all those who qualify for authorship are listed.

## CONFLICT OF INTEREST

None declared.

## Data Availability

The data that support the findings of this study are available from the corresponding author upon reasonable request.
